# Patients’ and physicians’ descriptions of occurrence and diagnosis of endometriosis: a qualitative study from Iran

**DOI:** 10.1186/1472-6874-14-103

**Published:** 2014-08-30

**Authors:** Hedyeh Riazi, Najmeh Tehranian, Saeideh Ziaei, Easa Mohammadi, Ebrahim Hajizadeh, Ali Montazeri

**Affiliations:** 1 Department of Reproductive Health and Midwifery, Faculty of Medical Sciences, Tarbiat Modares University, Tehran, Iran; 2 Department of Nursing, Faculty of Medical Sciences, Tarbiat Modares University, Tehran, Iran; 3 Department of Biostatistics, Faculty of Medical Sciences, Tarbiat Modares University, Tehran, Iran; 4 Mental Health research Group, Health Metrics Research Center, Iranian Institute for Health Sciences Research, ACECR, Tehran, Iran

**Keywords:** Endometriosis, Occurrence, Diagnosis, Iran, Qualitative research

## Abstract

**Background:**

The prevalence of endometriosis is considerable but its diagnosis is a dilemma. The aim of this study was to explore the perception and experiences of endometriosis patients and physicians about occurrence and diagnosis of endometriosis.

**Methods:**

A qualitative research using content analysis was used to obtain data from purposely selected endometriosis patients (12 participants) and gynecologists (6 participants) from January to September 2013 in Tehran. Data were coded and analyzed using a thematic approach.

**Results:**

Seven themes emerged: 1) pain localization, 2) Severity of pain and struggle for pain relief, 3) Feeling inability to play the role of femininity, 4) Reducing physical health, 5) Disruption of social life, 6) Looking for a reliable diagnostic indicator, 7) Uncertainty of physical examination. The results highlighted that patients with the disease can experience different feelings that interfere with their wellbeing and their lives, and sometimes could be disabling.

**Conclusion:**

Patients and physicians are looking for a certain, noninvasive and inexpensive diagnostic method. This study helps to promote clinical diagnostic view and knowledge development about description of endometriosis diagnosis to decrease diagnostic delay and mismanagement.

## Background

Endometriosis is a disease with considerable prevalence in communities. It has been estimated to affect 10% to 15% of women of reproductive age and 35% to 50% of women with pelvic pain, infertility, or both [1]. Women with endometriosis suffer from physical and mental symptoms that have an impact on all of their daily activities and quality of life [2-4]. A high hospital admission rate and surgical procedures and morbidity are reported in women with endometriosis [5]. Economic impact is another aspect of the disease. The annual financial burden of healthcare and productivity loss associated with endometriosis has been estimated at $2801 and $1023 per patient, respectively [6]. Recently Simeons et al. estimated that the total economic burden of the disease is €9579 per woman annually [7] which is equivalent to 418’219’140 Rails in Iran.

There are significant challenges in the disease diagnosis. Currently there is no reliable laboratory test or imaging technique that can detect the disease [4]. Thus, delay in diagnosis of endometriosis remains a problem [8] as it takes 8 to 11 years to be diagnosed [9]. The golden standard for the diagnosis are surgical procedures that are invasive and costly [10,11]. To develop non-surgical approaches for early detection of the disease, efforts are needed to recognize signs and symptoms of the endometriosis [12].

Most studies on endometriosis usually focus on issues related to either (i) clinical manifestations or, (ii) imaging techniques, or (iii) laboratory and surgical techniques, while it seems that to have a better understanding of the disease both experts’ and patients’ perspectives also are needed. There are few papers that report on patients’ experiences or clinicians’ perspectives about the disease diagnosis and they mostly come from western culture [13-16] or have limited focus on issues such as delay in diagnosis [17,18]. A qualitative study of 9 physicians and 41 patients found that pain explanation by the experts was incomplete compared with those of the patients [19]. Another study on the experiences of 20 Australian women diagnosed with endometriosis showed that physicians normalize the patients’ pain and patients cannot discriminate normal and abnormal menstruation. Furthermore patients deliberately conceal their menstrual problems for being free from stigmatization and all of these lead to diagnostic delay [18]. None of the studies focused on the concept of ‘occurrence and diagnosis’ of endometriosis. Thus, this study aimed to explore the topic in a qualitative fashion in order to have a greater and deeper understanding of endometriosis and perhaps contribute to the existing literature. In addition since many features of the disease depend on the cultural context, perceptions and attitudes, we thought a study from Iran might bring new insights from a different populations. It was hoped that this study could develop a new description of ‘occurrence and diagnosis’ of endometriosis.

## Methods

### Design

This was a qualitative study to explore the perception and experiences of patients and physicians about occurrence and diagnosis of endometriosis. To collect data semi structured face-to-face in-depth interviews were conducted from January to September 2013 in a teaching hospital affiliated to Tehran University of Medical Sciences.

### Sampling

The participants included both physicians and patients. The sampling continued until data saturation was reached. In all 18 individuals were interviewed (6 gynecologists and 12 patients). The only criterion for inclusion was a confirmed diagnosis of endometriosis for patients and having experience in endometriosis for physicians. The characteristics of participants showed maximum variances of samples. Sampling with maximum variation increases rigor of findings of qualitative studies [20]. Patients were aged between 22 and 37 years; having primary to higher educational level; living in different parts of Iran. Physicians had working experience from 5 to 27 years.

### Interviews

Data were collected using semi-structured interviews with patients and physicians. Most patients were asked the following questions: How did you realize your illness? Would you please describe your symptoms? Where did you go and what did you tell the doctor? What did they ask and what did they do for you? How did you feel? In the same way, most physicians were asked the following questions: Have you ever managed an endometriosis from the beginning to the end? How these patients express their problems? What do you do for diagnosis? To deepen the interviews such questions were asked: Is it possible to explain more? What do you mean? Why? How? Interviews were conducted in the hospital and lasted from 20 to 60 minutes. All interviews were carried out by H.R. and were recorded by using voice recorder and transcribed verbatim.

### Data analysis

Content analysis of transcribed data was done immediately after each interview. Data were coded and analyzed using a thematic approach [21]. The patient and physician’s perceptions and experiences about the diagnosis were considered. Each transcript was read for several times and the meaning units were diagnosed. After reviewing meaning units and encoding them, initial codes were developed. Data relating to each of the codes were then read and re-read, looking for similarities and differences and finally a total of 245 codes determined. Encoded data were minimized and compacted throughout the unit analysis process until the categories were identified. At the final step themes were developed from similar categories. An example of analysis process is shown in Table 1.

**Table 1:** An example of analysis process

**Theme**	**Category**	**Code**	**Story**
Feeling inability to play the role of femininity			
	Dyspareunia		
		- Complaint of pain during the coitus	- I suffered from painful sex; I couldn’t have sex anymore. I cried…
			- I felt a severe pain under my belly.
		- Disappearance of pain after the coitus	- …I had no pain after intercourse.
		Feeling of explosive pain during the coitus	- ….pain was so severe that I felt an explosive pain during sex.
		- Sexual disability	- …I couldn’t have different positions in sex due to my severe pain.
			- … when it took long I stopped. I could not tolerate it.
		- Burning sensation during the coitus	- … I could not tolerate it because I had burning sensation in my uterus.
	Infertility occurrence		
		- Complaint of infertility	- … I did not get pregnant after one year and it is very difficult for me to have a baby…
		- Ineffective infertility treatments	- …I had not successful IVF…
		- History of abortion	- I had an abortion two years ago…

#### Data credibility

To maintain data efficacy and credibility, manuscript was reviewed and modified with each unit analysis along with the extracted implications using supplementary participant views and suggestions. In addition, one qualitative researcher supervised and evaluated the entire study process.

#### Data validity

Interview transcripts and codes were presented to participants and they were asked if they agree with the meaning of the codes. In addition interviews were peer checked by academic colleagues who were not involved in the study to ensure the external validity.

### Ethics

The ethics committee of Terbiat Modares University approved the study (approval number 52/693). Participants signed an informed written consent. They were assured of their safety, anonymity and confidentiality. Refusal to take part in the study or withdrawal at any point posed no harm to participants. In order to ensure anonymity, participants’ number has been used in reporting the study.

## Results

Data analysis from interviews produced 245 codes, 26 categories, and 7 themes. The identified themes included: 1) Pain localization, 2) Severity of pain and struggle for pain relief, 3) Feeling inability to play the role of femininity, 4) Reducing physical health, 5) Disruption of social life, 6) Looking for a reliable diagnostic indicator and 7) Uncertainty of physical examination. Categories and themes are shown in Table 2.

**Table 2:** Themes and categories

**Themes**	**Categories**
Pain localization	
	Abdominal pain
	Headache
	Low back pain
	Anal pain
	Mastalgia
	Lower limb pain
Severity of pain and struggle for pain relief	
	Severity and type of pain
	Pain response to different actions
Feeling inability to play the role of femininity	
	Dyspareunia
	Infertility
Reducing physical health	
	Menstrual disturbances
	Digestive Disorders
	Urinary Disorders
	Complaint of irritating cyst
	Pelvic infection problems
Disruption of social life	
	Impairment in daily activities
	Emotional and communicational disturbances
Looking for a reliable diagnostic indicator	
	Association the symptoms with marriage
	Family history
	Use of ultrasound
	Use of laparoscopy
	Use of imaging techniques
	Use of biomarker
	Confusion in diagnosis
Uncertainty of physical examination	
	Feeling discomfort during examination
	Physical examination findings

### Theme 1: Pain localization

This theme shows that many patients with endometriosis suffer from various pains caused many problems for them. Discomfort and pain in different parts of the body and the sense of vulnerability, has been expressed by the majority of patients and physicians who have experienced various forms of it. This theme consists of 6 categories included: 1) abdominal pain, 2) headache, 3) low back pain, 4) anal pain, 5) mastalgia and 6) lower limb pain.

Pain is one of the main points repeatedly expressed by participants. These pains experienced with variety of intensity associated with menstrual period. Abdominal pain is almost existed, aggravated by menstrual period. Most physicians stressed that these patients refer due to painful menstrual periods and suffer from dysmenorrhea. One of the participants patient number 12 (pa12) said: “*I always had pain but in the menstrual period it was much more, Pain was so intense that I could not touch my belly”.*

Severe headache is another complaint which may feel stronger in early menstrual period but the important thing we have found is imagine the headache as a normal feature of menstrual period from physicians or lack of enough attention to it: *I had very severe headaches starting from behind my head and coming up to top of my head and it was very severe in the first and second day of menstruation but my doctor said: “it is normal” (pa8).* The other patient (pa2) said: *“10 days left to menstrual period I have been suffering from very severe headache causing blurred vision and nausea and I could not even get up because I would fell down and this pain continued for 24 hours but my doctor said: “It is premenstrual migraine and is normal””.*

Low back pain aggravated by menstruation was experienced with majority of patients: *There is no description for that pain. For example sometimes my belly and my lower back were pulling down. It seemed that something heavy heat my back drastically (pa11).* This pain May be present in the whole of the cycle but may be felt with greater intensity during menstruation.

Another complaint and fear of its vulnerability expressed was anal pain usually experienced as a pressure pain. It feels more when defecating and often increases during menstruation: *It's like a pressure on the anus and is a special pain. It was so unbearable that I wanted to scream (pa4).*

Some patients complained of mastalgia which is particularly reported pre-menstrual and sometimes this pain feels so severe that the patient cannot tolerate even her bra for example one of them said: *“I had a severe radiating pain in my nipples and I couldn’t even touch them” (pa8).*

Finally, lower limb pain is another complaint which concerns in this theme hurting the patients. This pain is sometimes experienced following dragging pain from abdomen or low back pain and in many cases the patient is suffering throughout the menstrual period. Some of patients described that they feel numbness or coldness in their feet. These findings showed that patients express discomfort from different and diffused pain.

### Theme 2: Severity of pain and struggle for pain relief

Two categories created this theme: 1) the severity and type of pain and 2) pain response to different actions.

Severity and type of pain imply the types such as intermittent and aggravated pain, chronic pain or severe and unbearable pain that was mentioned by nearly all cases. Patients described the pain and its severity in different types of descriptions for example: *I had terrible pain ...... It was unbearable (pa9).* Another patient said: *I was in such a severe pain that I couldn’t do anything, neither sitting nor standing , really nothing. It would get worse even if somebody would walk close to me. It was very very severe ……(pa5).* Some patients described it as explosive pain. This issue is also seen in the experience reported by physicians: *Some come with the chronic pelvic pain and pressure feel constantly (physician number 5 (phys5)).*

Patients have been seeking to find a solution to reduce pain, such as analgesics or steroids or nonpharmacological methods such as taking help of warmth and rest. Some participants mentioned that the pain was too severe to respond to conventional analgesics so intravenous analgesics and fluid therapy was needed to relieve pain. One of the patients said: *I felt that if I put something heavy on my belly it would reduce my pain. This would pacify me at least for a few minutes (pa9),* and patient number 5 said: *“My pain didn’t respond to even 2 or 3 Gelophens and only IV analgesic infusion could stop my pain”.*

These findings showed that patients tend to do anything for pain relief.

### Theme 3: Feeling inability to play the role of femininity

This study is conducted in a country that being a wife and being a mother makes women feel perfect and valuable. In other words, by performing the role of an spouse and ultimately being a mother, women will feel maturation and do realize themselves as a real woman. Disruptive factors which have interactions with these two important matters can cause problem in the role of femininity. Two categories created this theme: 1) dyspareunia and 2) infertility.

The majority of patients complained lower abdominal pain, severe itching during intercourse and inability to perform coitus. Many women endure the pain to be able to play their feminine role: *I suffered from painful sex; I wouldn’t be able to have sex. I cried. I felt a severe pain under my stomach. It was so severe that I couldn’t breathe at all. I just felt pain. I cried during intercourse but I had to bear the pain to make my partner happy (pa12).* Dyspareunia is so severe in some patients’ experiences that they feel they have an explosion in suprapubic region. Patients’ experiences show that the pain will go away after intercourse and only in rare occasions it will be continued for 24 hours.

Infertility is another factor which may interfere with sense of self as a woman. Many women realize that they cannot become pregnant following the disease: *In the first two years after marriage I did not want to get pregnant but when I wanted to get pregnant, I faced with infertility (pa3).* All the physicians mentioned that patients may refer to us due to infertility. The majority of patients refer to gynecologists due to an important problem which is infertility. Complaint of infertility and its ineffective treatments, increased incidence of miscarriage in case of pregnancy, are common problems. Since these two categories are important factors of interaction with the women’s roles of femininity so they created this theme.

### Theme 4: Reducing physical health

The main focus of this theme was to all categories which could interfere with physical health including: 1) menstrual disturbances, 2) Digestive Disorders, 3) Urinary Disorders, 4) Complaint of irritating cyst and 5) pelvic infection problems. Following these problems which involves various systems and divert patients from the road of health, finally, the patient feels that her health is reduced.

Irregularity and changing the pattern of menstrual cycles and spotting, result in feeling sick or feeling of disorder. Some patients complained of bimonthly menstruation or premenstrual or postmenstrual spotting which is mentioned by physicians too: *My bleeding was clotty and I had spotting from one week before menstruation (pa2).*

Digestive Disorders such as constipation or diarrhea, nausea, vomiting or bloody stools are some other kinds of problems, diminish health situation. Sometimes the patient is so critically ill that intolerance to food and water will appear. Digestive Disorders would be worse during the menstruation. One of participants expressed: “*In the first day of menstrual period, I was very sensitive to the smell of food just like a pregnant woman and even one spoon of food made me vomit. I did not eat food or even drink water for a day, I hated water. I would be nauseated after drinking water and I preferred not to drink at all, although I felt too thirsty” (pa9).*

Dysuria and hematuria are other disorders that can threaten the health of patients and majority of physicians had experienced it about endometriosis patients.

Ovarian cysts are another aspect of interfering health condition causing pain or other symptoms and are almost resistant to treatment: *I felt a severe pain in my whole abdomen that could not even breathe and my doctor diagnosed cysts by ultrasound.…(pa4).*

The last category of this theme is complaint of severe pelvic or gynecologic infections expressed by patients or physicians. Some patients complained of acute or chronic cervicitis and some others had severe infections resistant to treatment: *Each time I visited the doctor after marriage, he said that it was resistant pelvic infection; I was fine for 2 or 3 months with pills but would recur again (pa6).*

### Theme 5: Disruption of social life

Endometriosis can interfere with patient's lives and daily programs or communications. Two categories developed this theme: 1) impairment in daily activities and 2) Emotional and communicational disturbance.

Endometriosis negatively impacts on daily life and physical functioning. Severe disability in movement during the menstrual period, interference with religious practices and being upset from not doing religious duties are some of main issues found. Most patients have so much pain and discomfort that prevent them from daily activities. Based on most patient experiences this impairment is so much that makes them to be confined to bed and they are obliged to set their life plans considering the time of menstruation. Patients lose their ability in handling the affairs of life and feel that they need to be supported: *Pain was so intense that made me lie down in bed; I could not do anything even going to the bathroom was troublesome; I could not get up; I worked less; I cared less about my life; Every day I went to my mother’s house because she took care of me; she put hot towel on my belly or gave me my tablets ….(pa7).*

Feelings of hopelessness and fatigue, gloomy, feeling of depression, the need for support and problems in relationships with family members due to infertility are explained by majority of participants. Long term diagnosis is time and money consuming and in addition to psychological problems due to confusion and misdiagnosis, it can also disrupt order and routine of life. One of the patients stressed that: *I've been sick for a long time. I am so tired. I haven’t told anyone about my infertility. Everyone says “you have been married for five years, why do not you bear a baby?” I feel so bad and I cry all the time (pa6).*

### Theme 6: Looking for a reliable diagnostic indicator

Patients and physicians try to find a way for definite diagnosis. The seven categories identified in this theme were: 1) association the symptoms with marriage, 2) family history, 3) use of ultrasound, 4) use of laparoscopy, 5) use of imaging techniques, 6) use of biomarker and 7) confusion in diagnosis. These categories show the patients and physicians concerns about the best and the most reliable diagnostic tool.

The majority of patients mentioned that they had the symptoms before marriage. The experience of physicians implies some relation between these two: *Nulliparity and late marriage, sometimes make us more suspected about endometriosis (phys5).* Since virginity is very important factor in Iranian culture, sexual life starts after marriage and usually premarital sexual activity is not allowed. Thus, delayed marriage leads to late diagnosis of dyspareunia that is one of the most important symptoms of the disease. On the other hand it is believed that dysmenorrhea is normal and common during the virginity and it relieves after marriage. All of these may lead to diagnostic delay.

Existence of disease in other family members was stressed by many patients and physicians but negative family history does not rule out the disease.

Some of the diagnostic methods and imaging techniques which are used in this matter are ultrasound, MRI, CT scan or biomarkers explained by all the patients and physicians but laparoscopy is used as the golden standard for diagnosis. Some biomarkers such as CA125 are checked as a diagnostic indicator because we expect to be faced with an increase in its level. However sometimes in the presence of disease there isn't any rising or in case of rise it is related to other disorders.

Despite all the tests that are performed to diagnose endometriosis there are many uncertainties in the diagnosis. An incorrect diagnosis and incorrect treatment followed it may lead to mismanagement: *We thought that the pain is due to the Intestinal colitis. After four or five years of treatment I still suffered of pain and then my doctor showed concern about endometriosis (pa4).* Because these patients suffer from diverse and scattered pains, confusion in diagnosis is likely to happen. *I suffered from a severe headache but my doctor diagnosed the premenstrual migraine (pa2).* Patients with misdiagnosis of recurrent pelvic infections or chronic infections are treated for many years: *I had the pain in my lower belly and I had to visit doctors frequently and they all said that it was pelvic infection resistant to treatment (pa6).* Drug prescription, particularly analgesics or hormonal steroids in the absence of definite diagnosis, alleviates the symptoms temporarily, creates confusion in diagnosis and postpones definite diagnosis: *I was told that it was normal and you must tolerate it. They prescribed analgesics or oral contraceptive pills for me (pa9).*

It seems that although a definite diagnosis is the aim of patients and physicians, all the above-mentioned factors can delay the diagnosis.

### Theme 7: Uncertainty of physical examination

Information obtained through interviews with physicians and patients with endometriosis reflects the fact that physical examination is used as a diagnostic method but physicians stressed that they do not use it as a confident diagnostic method however sometimes existence of pain or adhesions or tumors or uterine fixation may help. Thus this theme emerged from two categories: 1) feeling discomfort during examination and 2) physical examination findings.

The majority of physicians stressed: “*…. However, the physical examination is helpful in diagnosis but do not have certainty” (phys1).* Some of the patients had feeling pain during examination: *During the examination following the pressure into the rectum I felt a terrible pain and screamed (pa4).* While some others had not mentioned it: *I had no problem and did not feel any pain during the examination (pa3).*

## Discussion

To the best of our knowledge, this is the first qualitative study about patients’ and physicians’ descriptions of occurrence and diagnosis of endometriosis in Iran. The results of this study describe the perception and experiences of occurrence and diagnosis of endometriosis from the perspective of 18 participants who took part in a qualitative study.

Based on the results emerged from content analysis and the themes; for the first time a description for occurrence and diagnosis of endometriosis was explored: *“Diagnosis of endometriosis is accompanied by perceptions and symptoms in the forms of expressing discomfort from the varied and diffused pain and a feeling of vulnerability and severe pain to the extent that it leads to struggle for relieving the pain in these patients. Another helpful condition in diagnosis of endometriosis is feeling of inability to play the role of femininity and feeling of reducing physical health and also disruption of social life which frequently refer patients to physicians; as a result physicians have been more careful and curious. Since physical examination findings are not reliable, physicians also look for a reliable diagnostic indicator such as laparoscopy which is golden standard”.* This description about endometriosis diagnosis is developed for the first time in Iran and is obtained from emerging themes and represents the patients’ and physicians’ experiences in order to understand and diagnosis of endometriosis. Most of studies have limited views of disease but we tried to have an expanded and generalized presentation. Nnoaham and coworkers have recently proposed a predicting model for endometriosis based on a multicenter research [22]. Findings of present study include almost all the components of their model, albeit we did not focus on stage of disease in this study. Other studies confirm these findings [23,24]. Pain is a symptom which is very substantial in these patients and almost all of them complained of it. Fauconnier and Chapron showed in their paper that painful symptoms are identified in endometriosis [25]. Besides endometriosis causes chronic pelvic pain symptoms [23,25]. Adhesion, inflammation and ovarian cysts are possible causes of pain. A common feature of these pains is worsening before and during the menstrual period. Dysmenorrhea is mentioned as the commonest symptom in some studies [23]. Sometimes dyschezia and dysuria are seen. Other studies also approved this finding [23,26]. Severe headache and mastalgia were some other kinds of pains found. Intensity, pattern and location of pain were varying and other studies showed this finding too [23]. In this study, patients described their pains as severe, unbearable, terrible and explosive. In Denny’s study, pain is described as intense or overwhelming [27]. Similar studies have found pain description as throbbing, gnawing, and dragging pain to the legs [28]. All of these indicate that in majority of cases pain is so severe that is intolerable. Therefore finding ways to educate medical professionals who may be reluctant to consider such pains as indication of an underlying pathology for endometriosis might play a helpful role in the diagnosis of disease [29].

Patients make every effort to reduce their pain. They use medical and nonmedical methods to relieve pain, for example using acupuncture [30]. In Cox’s study the main goal of women was to be drug free, but at the same time to get good pain management [31]. However elimination of pain is the principal concern of patients [10].

Infertility and dyspareunia are two significant factors interfering with the femininity of women living with endometriosis. They feel powerless to perform their family duties as a woman. Prevalence of infertility among women with endometriosis varies from 20% to 50% [32,33]. Nnoaham et al. cited sub-fertility due to blocked tubes [22]. Most women with endometriosis suffer from dyspareunia and infertility [28,32,34]. Living with dyspareunia has a significant negative impact on self-esteem and intimate relationship with partner, and on sexual functioning [35,36]. Infertility along with dyspareunia may lead to a reduced feeling of femininity. These findings are important to our understanding of endometriosis because previous studies did not focus enough on Asian or Iranian context. In Iranian culture procreation is a very important role for women and infertility is one of the causes of divorce. In addition, in Iranian culture infertility is a stigma and thus fertility is an important factor for family stability. It is argued that infertility in eastern culture might affect women more compared with women from western cultures [37]. Insecurity about fertility as well as distorted self-perception may contribute negatively to sexual satisfaction and sexual self-worth possibly by affecting self-esteem and female identity.

Disruption of social life was another finding of this study. Patients reported that they were unable to perform their daily activities due to pain and other complications of the disease. Similar findings were reported where reduced effectiveness and loss of work productivity were observed [27,38,39].

Although our purpose was not comparing patients’ and physicians’ views, physicians had less attention to social life, daily activities and communication issues. Even, Fauconnier et al. showed that different perception exist between clinicians and patients on issues such as pain [19]. Some aspects of endometriosis for example infertility may affect patient’s relationships profoundly. Denny’s study approved its effect on relationships with partners and family [27]. Disease has psychosocial impact on patients, so, in addition to physical; emotional and social aspects of their life must be considered too [4,17,34]. Prevalence of depressive and anxiety symptoms and also impaired quality of life are found in other studies [2,3]. Emotional well-being of patients is critically important. Seear found that empowering women reduces the stresses of the disease [40].

Results of our study indicate that patients and physicians try to find a way for definite diagnosis. Although signs of endometriosis may be found on physical examination but it is not dependable. Ultrasonography is sometimes effective especially in the case of endometriomas of the ovaries, but it is not an acceptable diagnostic tool [4]. CA125 may be elevated but not diagnostically reliable [4]. Family history of endometriosis may be a positive key leading to consider endometriosis [33]. It is necessary for physicians to have sufficient attention to patient complaints. Ignoring complaints or normalizing them is of common reasons causing delay in diagnosis [24]. So careful and detailed history-taking is necessary. Inappropriate diagnostic tests, misdiagnosis and mismanagement also may cause delay in diagnosis. Temporary relief of pain by analgesics or hormonal steroids creates confusion in diagnosis and postpones it. This is in line with other studies [17]. In Cox et al. study the worst experience of patients was trivialization of their disease [41]. Despite numerous attempts to find a non-invasive diagnostic method, laparoscopy is still the golden standard for the diagnosis of endometriosis [42].

The strengths of this study are that we have ensured a surgical diagnosis for all the women with endometriosis and almost all of them were newly diagnosed patients. Both physicians’ and patients’ participation and use of their experiences was another noticeable one. Arash hospital which we had sampling is a referral center and patients are referred there from all the cities of Iran. Moreover there is a cultural and religious attitude to the issue in present article. However, the findings of this study cannot be generalized across the endometriosis community because of cultural and religious effects.

## Conclusion

The study findings showed that perception of occurrence and diagnosis of endometriosis is rather a complex issue. However, both patients and physicians indicated that they were seeking for reliable diagnostic indicators. Perhaps the findings from this study could help to decrease the diagnostic delay in endometrosis.

## Competing interests

None of the authors have any conflicts of interest to report.

## Authors’ contributions

HR was the main investigator and carried out the study. NT and SZ supervised the study. EM was the study advisor and involved in data analysis. EH was the statistical advisor to the study. AM critically evaluated the manuscript and contributed to the writing process and prepared the final draft. All authors read and approved the manuscript.

## Pre-publication history

The pre-publication history for this paper can be accessed here:

http://www.biomedcentral.com/1472-6874/14/103/prepub
